# The use of the primary structure of the ITS1–ITS2 region for species identification in some submerged aquatic macrophytes of the genus Stuckenia

**DOI:** 10.18699/VJGB-23-21

**Published:** 2023-04

**Authors:** A.V. Mglinets, О.E. Kosterin

**Affiliations:** Institute of Cytology and Genetics of the Siberian Branch of the Russian Academy of Sciences, Novosibirsk, Russia; Institute of Cytology and Genetics of the Siberian Branch of the Russian Academy of Sciences, Novosibirsk, Russia

**Keywords:** Potamogetonaceae, Stuckenia, S. chakassiensis, S. macrocarpa, S. pectinata, S. vaginata, ITSI–ITS2 region, spices identification, Potamogetonaceae, Stuckenia, S. chakassiensis, S. macrocarpa, S. pectinata, S. vaginata, район ITS1–ITS2, идентификация видов

## Abstract

Applicability of ITS1–ITS2 primary structure for species attribution of representatives of the genus Stuckenia was experimentally tested. Analysis of the ITS1–ITS2 region sequences of S. vaginata and S. pectinata from public databases showed that they differed by insertions/deletions and single or double nucleotide substitutions. Besides, the ITS1–ITS2 region of S. pectinata was shown to be represented by two haplotype groups designated as S. pectinata type A and S. pectinata type B with good bootstrap support in phylogenetic reconstructions. In 28 samples identified as S. pectinata, S. vaginata, S. macrocarpa and S. chakassiensis on the basis of morphology, the ITS1–ITS2 region was sequenced in this study. Three groups of samples with good bootstrap support were revealed to be corresponding to S. vaginata, S. pectinata type A and S. pectinata type B. The S. vaginata group was formed by the samples identified on the basis of morphology as S. vaginata, and the S. pectinata type A group was formed by the samples identified on the basis of morphology as S. pectinata. The S. pectinata type B group was further divided into two subgroups, S. pectinata type B subgroup and S. chakassiensis subgroup. The S. chakassiensis subgroup included mainly the samples identified as such on the basis of morphology. The S. pectinata type B subgroup included samples identified on the basis of morphology as S. pectinata, S. vaginata and S. macrocarpa. We suppose that these samples were S. pectinata type B, S. macrocarpa and their hybrids.

## Abstract (ru)

Проведен биоинформационный анализ первичной структуры района ITS1–ITS2 образцов S. vaginata и S. pectinata, взятых из публичных баз данных, ссылки на которые приведены в опубликованных работах. Показано, что межвидовые различия S. vaginata и S. pectinata обусловлены делециями/вставками и одно- или двунуклеотидными заменами. Более того, вид S. pectinata по структуре района ITS1–ITS2 представлен двумя генотипами, которые обозначены как S. pectinata тип A и S. pectinata тип B, различия между которыми обусловлены одно- или двунуклеотидными заменами. Это демонстрирует возможность применения данного района для определения видовой принадлежности у представителей рода Stuckenia. Для экспериментальной проверки возможности использования данного района у 28 образцов, определенных на основании морфологических признаков как S. pectinata, S. vaginata, S. macrocarpa и S. chakassiensis, выполнено определение первичной структуры района ITS1–ITS2. Анализ полученных экспериментальных данных показал, что они распадаются на три группы, третья группа представлена двумя подгруппами. Эти группы соответствуют S. vaginata, S. pectinata тип A и S. pectinata тип B. В группу S. vaginata попали образцы, которые на основании морфологических признаков определены как S. vaginata. В группу S. pectinata тип A попали образцы, которые на основании морфологических признаков определены как S. pectinata. Группу S. pectinata тип B на основании первичной структуры района ITS1–ITS2 можно разделить на две подгруппы: S. pectinata тип B и S. chakassiensis. В подгруппу S. pectinata тип B вошли образцы, которые на основании морфологических признаков определены как S. pectinata, S. vaginata и S. macrocarpa. В подгруппу P. chakassiensis в основном вошли образцы, которые на основании морфологических признаков определены как S. chakassiensis. На основании выравнивания последовательностей подгруппы S. pectinata тип B сделано предположение, согласно которому в данную подгруппу объединены последовательности, принадлежащие S. pectinata тип B, S. macrocarpa и их гибридам.

## Introduction

Representatives of the genera Potamogeton L. and Stuckenia, formerly considered as a single genus Potamogeton, are aquatic plants present in all the continents except for the Antarctica. They inhabit both fresh and brackish standing and slow-moving waters. Both genera are characterized by high intraspecies morphologic variability causing difficulties for the systematics (Kaplan, Stepanek, 2003). Besides, there exist a lot of interspecies hybrids that are sometimes taken for individual species (Wiegleb, Kaplan, 1998). Another difficulty in the taxonomy of the genus is due to the existence of polyploids and aneuploids (Les, 1983; Hollingsworth et al., 1998; Kaplan, 2002; Fant et al., 2003; 2005; Lindqvist et al., 2006; Kaplan et al., 2009; Kaplan, 2010). According to literature data, the genus Potamogeton in the former broad sense counted about 1300 described species and interspecies hybrids, however, analysis of the herbarium samples allowed to identify only 69 to 90 species and 40 to 50 interspecies hybrids (Wiegleb, 1988; Wiegleb, Kaplan, 1998). In that sense, the genus was split in two subgenera Potamogeton L. and Coleogeton Rchb., species of the latter being distinguished by floating thickened leaves with long sheaths, hydrophilic (not anemophilic) inflorescences on long peduncles, commonly bearing widely separated whorls of flowers, as well as characteristic pollen structure (Sorsa, 1988). In the species of the subgenus Potamogeton, chromosome number varies from 2n = 14 to 2n = 52, while in the species of the subgenus Coleogeton it is 2n = 78 (Les, 1983; Les, Haynes, 1996). The distinction of these two subgenera was supported by the complete absence of hybrids between species of these subgenera, while within both subgenera hybridisation is quite widespread (Tsvelev, 1996; Wiegleb, Kaplan, 1998). Due to a number of reasons, Coleogetоn was proposed to be considered as a separate genus (Les, Haynes, 1996). Of the names suggested at the generic rank, Coleogetоn and Stuckenia Börner, the latter is correct (Holub, 1997; Haynes et al., 1998a, b). At present, there exist two parallel versions of species names, for example, Potamogeton pectinatus L. is a synonym of Stuckenia pectinata (L.) Börner and so on. In this work we will consider the taxon in question at the generic level, as Stuckenia, and will use the specific names even if the cited authors used Potamogeton.

Studies of phylogenetic relationships in the family Potamogetonaceae, including representatives of the genera Potamogeton and Stuckenia using both plastid DNA markers (Iida et al., 2004) and 5S-NTS region of the nuclear genome (Lindqvist et al., 2006) showed that members of these genera cluster into two clearly distinguishable groups with high bootstrap support. This is in good accordance with conclusions made on the basis of morphologic characters. However, Q.D. Wang et al. (2007) found the latter region of Potamogeton and Stuckenia similar and did not support separation of the latter genus.

Taxonomy of the genera Potamogeton and Stuckenia, as well as other aquatic plants, is based mainly on anatomy and morphology of leaves, fruits, stems. The study of herbarium specimens showed that these characters are highly variable within a species. On the whole, all species of these two genera can be divided into three groups according to the degree of variability: (i) species with rather uniform morphological traits in spite of wide geographic range, their species attribution does not cause difficulty (P. obtusifolius Mert. et Koch, P. praelongus Wulf., P. crispus L.); (ii) species with a wide spectrum of variability within geographic range, so they can be sometimes misidentified as novel species or interspecies hybrids (P. striatus Ruiz. et Pav., S. filiformis Pers. and others); (iii) species with extremely high morphologic variability even in the same area so that their species attribution is always problematic (S. pectinata and others) (Wiegleb, 1988). Experimental cultivation of the clones of different species under controlled conditions (at different depths, different nutritional values of the substrate, different illumination) showed that morphological traits essential for the taxonomy vary with environmental changes and therefore cannot serve as reliable markers for species attribution (Kaplan, 2002). For example, herbarium samples collected in Central Russia, in the Caucasus, Middle Asia, Southern Siberia identified as S. filiformis upon re-examination turned out to be S. pectinata (Maemets, 1979). It has been noted that in the Arctic region of the European part of the former USSR, S. filiformis as well as interspecies hybrid S. filiformis Pers. × S. vaginatus Turcz. are often identified as S. pectinata (Maemets, 1979). Taxonomic revision of the Stuckenia species also revealed cases of erroneous species attribution in the group considered (Kaplan, 2008).

In the late 20th century biochemical markers, first of all, isozymes came into use for the study of the representatives of the genus Potamogeton and Stuckenia. These markers were used for the study of presumed interspecies hybrids considered as such on the basis of morphology (Hollingsworth et al., 1996; Kaplan, Stepanek, 2003). Later on, methods of molecular biology (RAPD, PCR RLFP, AFLP analyses) were employed for the study of interspecies hybrids (Whittall et al., 2004; Uehara et al., 2006; Kaplan et al., 2009).

The primary structure of the ITS1–ITS2 region of the nuclear genome is widely used for the study of phylogenetic relationships of a broad spectrum of organisms. At the same time, it can be utilized for species attribution of a given specimen when other approaches are inapplicable or complicated (Kress et al., 2005; Fazekas et al., 2012). This is the principle of the method of DNA barcoding of living organisms. However, this approach has certain limitations which should be considered in its practical applications and which are widely debated in literature (Shneyer, Rodionov, 2019). Since a large body of biodiversity remains poorly studied, primary structure of the ITS1–ITS2 region allows to make a conjecture about existence of new species but not isolate and describe them (Desalle, 2006).

The aim of the present work was to study the applicability of the primary structure of the ITS1–ITS2 region for species attribution of a number of samples of the genus Stuckenia, classified on the basis of morphology as S. pectinata, S. vaginata, S. macrocarpa (Dobroch.) and S. chakassiensis (Kashina) Volobaev

## Materials and methods

The present study is based on sequences of both reliably identified species present in Gene Bank at the moment of this study and original sequences from 28 plant specimens. Of these, 7 were identified as S. pectinata, 7 as S. vaginata, 9 as S. macrocarpa and 5 as S. chakassiensis on the basis of morphology (Suppl. Material1). Plant material has been provided by L.M. Kipriyanova (Institute for Water and Environmental Problems of Siberian Branch of the Russian Academy of Sciences, Novosibirsk department, Russia). DNA was extracted from dry (herbarium) material or fixed and stored in ethanol. DNA extraction was performed with the use of 2x CTAB buffer as described by S.O. Rogers and A.J. Bendich with modifications (1985). Plant tissue (0.02–0.05 g of dry or 0.2–0.3 g of stored in ethanol) was thoroughly grinded in a mortar in the presence of 0.05 g aluminium oxide and 1 ml of extraction buffer freshly prepared before the extraction procedure dissolving 0.03 g polyethylene glycol 6000 and 0.05 g dithiothreitol in 1 ml 2х СТАВ (2 % СТАВ, 1.4М NaCl, 0.1М TRIS pH = 8.0, 20 mM EDTA). Homogenate was transferred to 2 ml tubes and incubated for 30 min at 75 °С. Then, 1 ml dichloromethane was added to each tube and thoroughly mixed for 10 min, the tubes were centrifuged for 10 min at 6708 x g. The supernatant was transferred to a fresh tube and added with 0.2 volumes of 5х СТАВ (5 % СТАВ, 350 mM EDTA), mixed and incubated for 10 min at 65 °С. Then each tube was added with 1 ml dichloromethane, mixed for 10 min and centrifuged as described above. The supernatant was transferred to fresh tubes and DNA was precipitated adding equal volume of isopropanol, mixing and keeping at –20 °С for 1 h or more. Nucleic acids were precipitated by centrifuging as described above, washed twice with 70 % ethanol, dried and resuspended in 50 μl deionised water. For Polymerase Chain Reaction (PCR) 10-fold dilution (1 part nucleic acid solution: 9 parts of water) was used.

Supplementary Materials are available in the online version of the paper: https://vavilovj-icg.ru/download/pict-2023-27/appx4.pdf


PCR reaction was performed in a volume of 20μl with 2 μl of 10х ammonium-sulphate buffer, 2 μl of 25 mM MgCl2, 0.2 μl of the Tаq polymerase (5 U/μl), 0.15 μl BSA (10 mg/ ml), 1 μl of forward and reverse primers (10 pM) each, and 2 μl of diluted DNA. Concentration of dNTPs in the reaction mixture was 0.2 mM each. PCR reaction was held under following conditions: initial denaturation 95 °С – 3 min; then 38 cycles including: denaturation at 94 °С – 30 s, primer annealing at 58 °С – 30 s, elongation at 72 °С – 60 s; terminal elongation at 72 °С – 5 min. To amplify the ITS1–ITS2 region, ITS-5m (5′-GGAAGGAGAAGTCGTAACAAGG) and ITS-4 (5′-TCCTCCGCTTATTGATATGC) primers were used (Sang et al., 1995).

For the sequencing reaction, the same primers were used as for amplification. In some cases, when the use of the primers ITS-5m and ITS-4 failed to produce chromatograms of the suitable quality, specially designed sequencing primers were used: seq-ITS-F (5′-GATGACTCTCGGCAACGG ATA) and seq-ITS-R (5′-CTCGATGGTTCACGGGATTCT). Sanger sequencing reaction was performed with the use of ABI PRISM® BigDye™ Terminator v3.1 Ready Reaction Cycle Sequencing Kit. Determination of the primary structure of the resulting products was done at the SB RAS Genomics Core Facility (Novosibirsk). Nucleotide sequences obtained in this study were deposited in GenBank under accession numbers MH427614 to MH427641

Besides, sequences HE613425, HE613426, HE613427, HE613428, HE613433, HE613434, KF270926, KF270927, KF270928 and KF270929 were taken from public data-bases.

Sequences were aligned by ClustalW program incorporated into Mega 5 package (Thompson et al., 1994; Tamura et al., 2011). Estimations of pairwise divergence between sequences were conducted in MEGA 5 (Tamura et al., 2011). The trees were constructed by the Maximum Likelihood method based on the Tamura–Nei model by means of MEGA 5 package (Tamura, Nei, 1993). Numbers at the nodes represent bootstrap values as percentages out of 1000 replicates and are shown only for values greater than 50 %

## Results

Study of applicability of the ITS1–ITS2 region for species identification on the basis of sequences from public databases

To make sure that data on the primary structure of the ITS1– ITS2 region are applicable for species identification of the representatives of the genus Stuckenia, the following sequences were analysed: НЕ613433, НЕ613434, КF270928, КF270929 referred to as belonging to S. vaginata, and KF270926, HE613427, КF270927 НЕ613425, НЕ613426 НЕ613428, attributed to S. pectinata (McMullan et al., 2011; Kaplan et al., 2013). These sequences were obtained by two independent research teams who sequenced the ITS1–ITS2 region both in S. pectinata, and S. vaginata. The entries beginning with “КF” come from the Institute of Botany, Academy of Sciences of Czech Republic, and those beginning with “НЕ” were obtained by a research group from Great Britain. An analysis of the origin of the specimen studied showed that they were collected in rather distant geographic points. Namely, S. vaginata specimens were sampled in the Bothnia Bay near the coast of Sweden and Finland, in the Irkutsk region (Russia), and in the USA. S. pectinata samples were collected in USA, Netherlands, Great Britain, Italy, Russia and India.

Pairwise comparison of the above mentioned sequences showed that intraspecies differences within S. pectinata revealed in one of the studies coincided with those revealed in the other study (Table 1). Some ITS1–ITS2 sequences of S. pectinata obtained in different studies were identical. Pairwise comparison of ITS1–ITS2 sequence of S. vaginata demonstrated similar results. Thus, the data on the ITS1–ITS2 primary structure of a species obtained in one study are supported by those of the other study

**Table 1.**
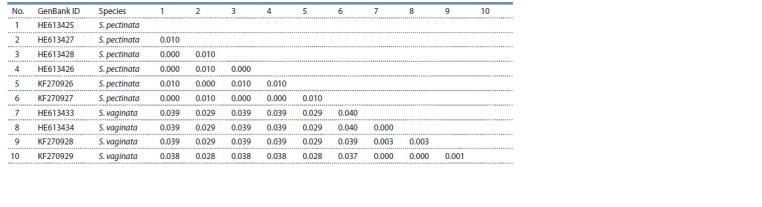
Matrix of pairwise uncorrected p-distances of the concatenated sequences of the ITS1–ITS2 region of S. pectinata and S. vaginata of different provenance, taken from GenBank

**Fig. 1.**
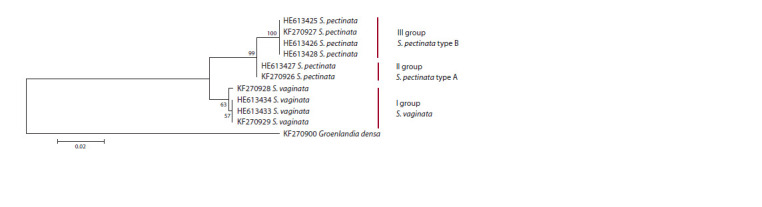
Maximum likelihood phylogenetic tree constructed on the basis of the primary structure of the ITS1–ITS2 region of the representatives of S. vaginata and S. pectinata from public databases.

The sequences taken from public databases were used to construct a phylogenetic tree (Fig. 1). This tree contains three rather well supported groups. The entire group I is formed by the sequences referring to S. vaginata while groups II and III are formed by the sequences referring to S. pectinata.

All specimens belonging to the groups II and III were identified as S. pectinata, but had different structure of the ITS1–ITS2 region, this difference being supported by high bootstrap values. To distinguish between the genotypes within S. pectinata samples, those from group II were denoted as S. pectinata type А (S. pectinata genotype А), and those from group III – as S. pectinata type В (S. pectinata genotype В). Table 2 presents the alignments of the sequences of the three indicated groups. It can be seen that S. pectinata type А is represented by two haplotypes differing by a deletion at the position 136 and 138 (KF270926). Other samples of S. vaginata and S. pectinata type В have no such deletion. This haplotype with the deletion was not found in the samples studied in the present work, so this unique variant is not considered further. As seen from the alignments, S. vaginata differs from S. pectinata type А and S. pectinata type В by three indels (two one-nucleotide and one nine-nucleotide) and several nucleotide substitutions (sixteen one-nucleotide and two two-nucleotide). The differences between S. pectinata type А and S. pectinata type В are smaller and consist of five one-nucleotide and one two-nucleotide substitutions. Thus, the primary structure of the ITS1–ITS2 region not only allows to identify known species S. vaginata and S. pectinata but also reveals the hitherto unknown type dichotomy of the latter for А and В types.

**Table 2.**
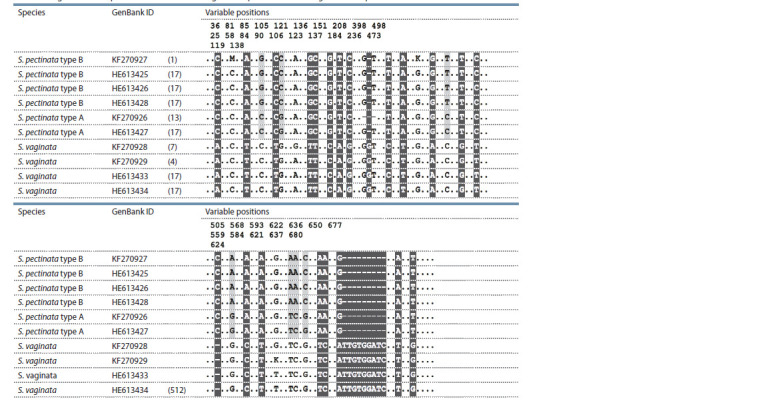
Alignment of sequences of the ITS1–ITS2 region of S. pectinata and S. vaginata from public databases Note. Positions differing between S. vaginata and S. pectinata are marked with black, between S. pectinata type A and S. pectinata type B are marked with grey.

Sequencing and analysis of the ITS1–ITS2 region in the representatives of the genus Stuckenia

Primary structure of the ITS1–ITS2 region was determined in 28 samples (see Suppl. Material). Also, three sequences from public databases were involved into analysis to provide a reference: HE613427 representing “S. pectinata type А”, HE613428 representing “S pectinata type В” and HE613434 representing “S. vaginata”. These sequences served as references to make species attribution of the sequenced samples to S. vaginata, S. pectinata type А or S. pectinata type В according to the primary structure of the ITS1–ITS2 region.

**Fig. 2.**
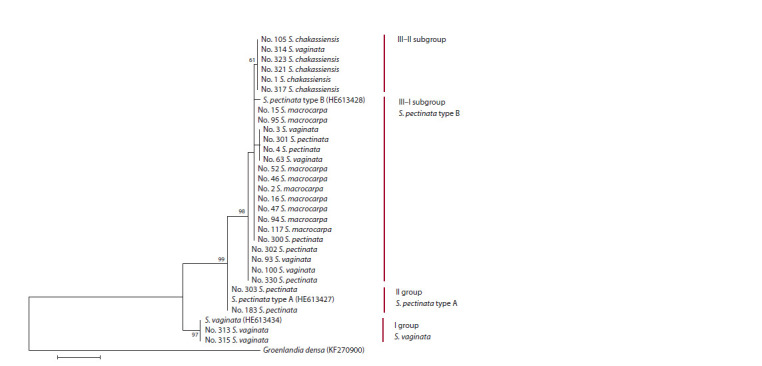
Maximum likelihood tree reconstructed on the basis of the primary structure of the ITS1–ITS2 region in the samples with species attribution according to morphology (in combinations with the generic names as in the data source).

The so formed data array was used to reconstruct a phylogenetic tree (Fig. 2). The sequences formed three groups with good bootstrap support. The first group was formed by 2 specimens (Nos. 313 and 315) with the ITS1–ITS2 region typical of S. vaginata. On the basis of morphology, they were also classified as S. vaginata. The second group was formed by 2 specimens (Nos. 183 and 303) with the ITS1–ITS2 region of S. pectinata type A. On the basis of morphology, they were also classified as S. pectinata. The third group could be separated into two subgroups – III–I and III–II. The subgroup III–I was formed by 18 specimens with the ITS1–ITS2 region of S. pectinata type B. On the basis of morphology, 9 specimens (Nos. 2, 15, 16, 46, 47, 52, 94, 95 and 117) were classified as S. macrocarpa, 5 – as S. pectinata (Nos. 4, 300, 301, 302 and 330), 4 – as S. vaginata (Nos. 3, 63, 93 and 100). The subgroup III–II was formed by 6 specimens. On the basis of morphology, 5 specimens (Nos. 1, 105, 317, 321 and 323) were classified as S. chakassiensis, 1 – as S. vaginata (No. 314).

Alignment of the sequences belonging to the subgroups III– I and III–II is given in Table 3. According to the nucleotides in the positions 102–103, the sequences of the subgroup III–I formed three clearly distinguishable batches. The first batch contained sequences from the samples Nos. 3, 63, 93, 100, 301, 302, 330 and the reference sequence HE613428. The second batch contained sequences from the samples Nos. 2, 46, 47, 52, 94, 95,117, and 300. The third batch contained sequences from the samples Nos. 4, 15 and 16.

**Table 3.**
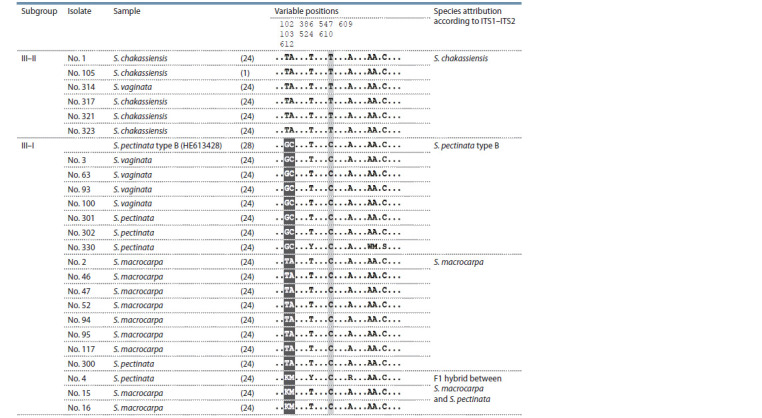
Alignment of sequences of the ITS1–ITS2 region of the samples from III–I and III–II subgroups Note. Positions differing between S. pectinata type B and P. macrocarpus are marked with black, differing P. chakassiensis from S. pectinata type B and P. macrocarpus are marked with grey.

The samples Nos. 3, 63, 93 and 100 belonging to the first of the mentioned batch were identified as S. vaginata, the samples No. 302 and 301 with the same primary structure of the ITS1–ITS2 region were identified as S. pectinata type B. Identical primary structure was shared by the reference sequence HE613428. The sequence of the sample No. 330 differed from that of the above mentioned samples by a number of polymorphic positions (seen in the sequencing chromatograms as superimposed peaks) not found in the other samples, and in the positions 102–103 it had the same nucleotide composition as the samples Nos. 3, 63, 93, 100, 301, 302 and HE613428. Thus, this batch was formed by the samples identified on the basis of morphology as S. vaginata and S. pectinata, although on the basis of the primary structure of the ITS1–ITS2 region these samples should be classified as belonging to S. pectinata type B. Such discrepancy of the morphologic and molecular data could result from misidentification. Earlier in the comparative investigation of S. vaginata and S. pectinata, it was shown that S. pectinata had two recognition sites for the restrictase CfoI (GCGC) in the ITS1–ITS2 region, while S. vaginata had only one such site (King et al., 2001). In our data array, the samples Nos. 3, 63, 93, 100 and 314 have two recognition sites, which is typical of S. pectinata. Therefore, it is highly probable that species attribution of the samples Nos. 3, 63, 93 and 100 was erroneous and they should be considered as S. pectinata. Thus, it may be stated with a high degree of confidence that the first batch of the primary structure of the ITS1–ITS2 region is composed by the samples of S. pectinata, or more precisely, S. pectinata type B.

Out of 8 samples of the second batch, 7 (Nos. 2, 46, 47, 52, 94, 95 and 117), were identified on the basis of morphology as S. macrocarpa and 1 (No. 300) – as S. pectinata. Since all sequences of this batch are identical, it is probable that the sample No. 300 was misidentified, and the second batch is composed by the samples of S. macrocarpa

The sequences composing the third batch (samples Nos. 4, 15 and 16) were identical and characterized by heterogeneity for the positions 102 and 103. Position 102 contained both G, as in S. pectinata type B, and T, as in S. macrocarpa. Position 103 contained both C, as in S. pectinata type B, and A, as in S. macrocarpa. Thus, these samples may be considered as interspecies hybrids between S. pectinata type B and S. macrocarpa. According to morphologic traits, the samples Nos. 15 and 16 were identified as S. macrocarpa, and the sample No. 4 – as S. pectinata.

Six samples forming the subgroup Ш–II had identical ITS1–ITS2 region, 5 of them, according to morphology, had been identified as S. chakassiensis, and one – as S. vaginata (sample No. 314). However, it is highly improbable that it really represents S. vaginata, since the structure of its ITS1– ITS2 region, in particular, the presence of two recognition sites for the CfoI restrictase is not typical of S. vaginata. If this sample is excluded from consideration, the subgroup Ш-II is constituted by the samples identified on the basis of morphology as P. chakassiensis. Therefore, it can be supposed that this entire subgroup is formed by representatives of the latter species. As seen from the alignments of the sequences of the ITS1–ITS2 region of the samples belonging to the subgroups III–I and III–II (see Table 3), the only difference between the mentioned subgroups consists in one nucleotide substitution, T/C in the position 524. Thus, according to the nucleotide in this position, samples of S. chakassiensis can be unequivocally identified by the primary structure of the ITS1–ITS2 region.

## Discussion

As noted above, the growing of representatives of the genus Potamogeton and Stuckenia under different ecologic conditions showed that a large part of morphologic traits basic for the taxonomy of the genus varies along with the growth conditions (Kaplan, 2002). Since the majority of investigators describe new taxa based solely on morphologic traits without any study as to the stability of their manifestation in different environments, ecological modifications were often described as new species (Kaplan, 2002). Thus, morphologic traits turned to be not too reliable for species identification in pondweeds, and there arises a need for developing markers applicable for species identification in the genus Stuckenia. Primary structure of the ITS1–ITS2 region is suggested here as a suitable marker for this purpose.

An analysis of the sequences of the ITS1–ITS2 region of S. vaginata and S. pectinata from public databases showed that these sequences differed, thus making the primary structure of this region a promising marker with respect to identification of the mentioned species. Moreover, two genotypes of this region were revealed in S. pectinata, designated as S. pectinata type A and S. pectinata type B.

These results were used to analyze the region ITS1–ITS2 in 28 samples of the genus Stuckenia, identified on the basis of morphology as S. vaginata, S. pectinata, S. chakassiensis and S. macrocarpa. Out of seven samples classified as S. vaginata, only two could be unequivocally attributed to this species according to the primary structure of their ITS1–ITS2 region, while the other five samples according to the primary structure of the studied region fit S. pectinata more. Such discrepancy of species attribution made on the basis of morphologic traits and molecular data may be due to original misidentification of the studied samples. Two samples classified as S. vaginata have only one recognition site for the CfoI restrictase in their ITS1–ITS2 region that is typical of S. vaginata, and rest of the samples classified as S. vaginata have two such recognition sites, which is typical of S. pectinata. This example demonstrates that species attribution made solely on the basis of morphology does not guarantee correct species identification, for the purpose of which other approaches should be supplemented, in particular, the use of molecular data on the ITS1–ITS2 region appears to be appropriate.

The botanical assessment of representatives of the genus Stuckenia showed that S. pectinata is a polymorphic species and even an opinion that it might be a composite species was put forward, although no evidence was provided (Maemets, 1979; Kashina, 1988). Our result revealing the existence of two groups of the primary structure of the ITS1–ITS2 region in S. pectinata (S. pectinata type A and S. pectinata type B) favours the view that S. pectinata includes several species that are indistinguishable or hardly distinguishable at the level of morphology but clearly differ at the level of the ITS1–ITS2 region. Also, it should be noted that no intermediate forms between A and B type of S. pectinata were revealed. The existence of two cryptic species hidden under the name S. pectinata supposed on the basis of the structure of the ITS1–ITS2 region is supported by literature data coming from the RAPD analysis of S. pectinata samples of different origin (Mader et al., 1998). The RAPD spectra of the samples from the River delta differed from those of the samples collected in Italy, Germany, Poland, France and the Saint-Petersburg surroundings. The samples from Spain and Egypt also differed from each other as well as from the above mentioned ones. P.A. Volkova et al. (2017), who studied the same ITS1–ITS2 region, revealed its uniformity in Europe but high differentiation in southern Siberia. All this allows to suppose that there exist at least two and perhaps more “forms” of S. pectinata. Their phylogenetic relationships are clear, but their taxonomic status is obscure. Whether they represent cryptic species, subspecies or merely intraspecies polymorphism requires further investigation. 

Some of the samples analysed in the present work have been classified as S. chakassiensis. In the phylogenetic tree (see Fig. 2), these samples formed a separate subgroup. These samples were categorized as a subgroup because the bootstrap value did not permit to consider them as a separate group. The primary structure of the ITS1–ITS2 region was identical in all representatives of the subgroup, it was also identical in a sample classified on the basis of morphology as S. vaginata and discarded as such according to the structure of the ITS1–ITS2 region. This allows to suppose that the species S. chakassiensis really exists although investigators can experience difficulties in its identification. At the same time, the data on the primary structure of the ITS1–ITS2 region allows to identify this species. P.A. Volkova et al. (2017) studied the same ITS1–ITS2 region and also the plastid rpl32-trnL spacer and found no correspondence between the sequence data and diagnostic morphological character of S. chakassiensis (which is not as convincing, being the presence of sclerenchyma strands in leaves). Actually, our data evidence for the same with respect to the above mentioned specimen identified as S. vaginatus. Taken together, the results of P.A. Volkova et al. (2017) and of the present study can be interpreted such that the species S. chakassiensis does exist but its only diagnostic morphological character proposed is unreliable and may lead to misidentifications

The existence of S. chakassiensis and its difference from S. pectinata is indirectly supported by the data coming from the study of metal contents in pondweeds and common reed (Phragmites australis Trin. ex Steud) from brackish lake Shira and freshwater reservoir Bugach (Ivanova et al., 2015). Differences in the contents of metals in the plants from different water bodies were shown for pondweeds but not for the common reed. At the same time, pondweeds collected in a desalinated part of Lake Shira did not differ from those collected in more salty water of the same lake. These paradoxical results can be easily interpreted if to suppose that the pondweed from the Shira Lake, with accordance to our data, belonged to S. chakassiensis, while the pondweed from the Bugach reservoir represented S. pectinata, that is, in fact, two species have been mixed. One of them grows in salt water, while the other in fresh or brackish water. In contrast to the pondweeds, common reed is adapted to the growth in fresh and brackish water as well as salt water, both studied lakes harbor the same species, the populations of which do not differ in metal contents. It should be noted that mineralization in Lake Shira is 15.9 g/l (Guseva et al., 2012). This is above the limit of the level of salinity that plants of S. pectinata withstand, over which their death begins (Coffey, 2001).

Especially interesting are the samples from the subgroup III–I classified on the basis of morphology as S. vaginata, S. pectinata and S. macrocarpa. According to the primary structure of the ITS1–ITS2 region, they can be categorized into three batches. The first of them is composed by samples that harbor the GC dinucleotide in the positions 102–103 (see Table 3); this is a characteristic of the reference sequence HE613428, S. pectinata type B. The sequences from the second batch harbor TA in these positions; the majority of the samples from this batch were classified as S. macrocarpa on the basis of morphology. The last batch is composed by the samples which harbor both G and T in the position 102 and both C and A in the position103. That is, the third batch can be obtained by a mixture of any sequence from the first batch with any sequence from the second one. This allows to suppose on the basis of the molecular data on the primary structure of the ITS1–ITS2 region that the III–I subgroup is composed by the samples of S. pectinata type B, S. macrocarpa and interspecies hybrids between S. pectinata type B and S. macrocarpa. This can be experimentally tested by comparative analysis of morphology and anatomy of presumed original species and their hybrids. If molecular data find support in the morphology, this may be interpreted as evidence for the existence of S. macrocarpa as a separate species. Earlier, P.A. Volkova et al. (2017) also obtained somewhat confusing molecular results with respect to three analysed specimens morphologically identified as S. macrocarpa: they shared a specific ITS1–ITS2 haplotype but had a haplotype of the rpl32-trnL spacer found also in three other species.

In conclusion, the data obtained in the present work demonstrate the applicability of the primary structure of the ITS1–ITS2 region for species attribution and revealing species misidentification in the genus Stuckenia, which in some cases may be more reliable than morphological data.

## Conflict of interest

The authors declare no conflict of interest.
